# Biochemical and Morphological Characteristics of Some Macrofungi Grown Naturally

**DOI:** 10.3390/jof7100851

**Published:** 2021-10-12

**Authors:** Ezelhan Selem, Yekbun Alp, Suat Sensoy, Yusuf Uzun, Seyda Cavusoglu, Neva Karatas, Sezai Ercisli, Nurettin Yilmaz, Halina Ekiert, Hosam O. Elansary, Agnieszka Szopa

**Affiliations:** 1 Field Crop Department, Institute of Natural and Applied Sciences, Van Yuzuncu Yil University, Van 65080, Turkey; ezelhanselem@hotmail.com; 2 Horticultural Sciences, Institute of Natural and Applied Sciences, Van Yuzuncu Yil University, Van 65080, Turkey; yekbunalp@hotmail.com (Y.A.); nrtnylmz47@gmail.com (N.Y.); 3 Department of Horticulture, Faculty of Agriculture, Van Yuzuncu Yil University, Van 65080, Turkey; suatsensoy@yyu.edu.tr (S.S.); scavusoglu@yyu.edu.tr (S.C.); 4 Department of Pharmaceutical Sciences, Faculty of Pharmacy, Van Yuzuncu Yil University, Van 65080, Turkey; y.uzun@yyu.edu.tr; 5 Department of Nutrition and Dietetics, Faculty of Health Sciences, Ataturk University, Erzurum 25240, Turkey; ngungor@atauni.edu.tr; 6 Department of Horticulture, Faculty of Agriculture, Atatürk University, Erzurum 25240, Turkey; 7 Department of Pharmaceutical Botany, Faculty of Pharmacy, Medical College, Jagiellonian University, Medyczna 9, 30-688 Kraków, Poland; halina.ekiert@uj.edu.pl (H.E.); a.szopa@uj.edu.pl (A.S.); 8 Plant Production Department, College of Food and Agricultural Sciences, King Saud University, Riyadh 11451, Saudi Arabia; helansary@ksu.edu.sa

**Keywords:** macro-fungi, soluble solid content, total antioxidant capacity, total phenolic contents, Van, Turkey

## Abstract

Recently, the production of macro-fungi (mushrooms) has steadily increased, and so has their economic value, in global terms. The use of functional foods, dietary supplements, and traditional medicines derived from macro-fungi is increasing as they have numerous health benefits as well as abundant nutrients. This study aimed to determine some biochemical contents (pH, soluble solid contents (SSC), total antioxidant capacity (TAC) and total phenolic contents (TPC)) of eight edible macro-fungi species growing naturally (in the wild) in Turkey. The samples were collected in the Van Yuzuncu Yil University (VAN YYU) campus area in the months of April–May 2018, in different locations, and brought to the laboratory, and the necessary mycological techniques were applied for their identification. Location, habitats, collection dates and some morphological measurements were determined for all identified species. Biochemical parameters of the macro-fungi species were analyzed separately both in cap and stem. The color values (L, a, b, Chroma and hue) were separately evaluated on cap surface, cap basement and stem. Results showed that there were significant differences for most of the biochemical parameters in different organs between and within species. The pH, SSC, TAC and TPC values varied from 6.62 to 8.75, 2.25 to 5.80° brix, 15.72 to 57.67 TE mg^−1^ and 13.85 to 60.16 gallic acid equivalent (GAE) fresh weight basis. As a result of the study, it was concluded that the parameters such as total antioxidant capacity, total phenolic content and soluble content in *Morchella esculenta*, *Helvella leucopus*, *Agaricus bitorquis* and *Suillus collinitus* were higher than for the other species and clearly implied that they may be further exploited as functional ingredients in the composition of innovative food products.

## 1. Introduction

Macro-fungi (Mushrooms) commonly spread on the earth, grow naturally in diverse habitats and have been seen as an important food source by mankind for centuries. They are also widely used in medical, pharmaceutical, cosmetic and commercial applications due to their valuable metabolic content [1]. Macro-fungi, known as a great source of food, are rich in terms of protein, fiber, vitamins, minerals and many different nutrients, while they have low carbohydrates and fat content [2,3,4,5,6,7,8,9]. Naturally grown macro-fungi species have different morphological traits and biochemical content and are very important in terms of both biodiversity and human consumption. Due to the poisoning cases that occur as a result of unconscious consumption in our country and the world, the features of macro-fungi species should be well known. Therefore, it is crucial to identify the macro-fungi and to determine whether they are edible or non-edible. With the understanding of the nutritional values of mushrooms, the number of studies on this subject has increased rapidly, and macro-fungi have been shown to be rich in phenolic compounds and natural antioxidants [10,11,12,13,14].

In the last two decades, the health-promoting properties of different plants, in particular naturally grown ones, have attracted the attention of consumers and the food industry. In fact, macro-fungi do not constitute a significant portion of the human diet, and their consumption continues to increase mainly due to their functional benefits attributed to the presence of bioactive compounds which may act as antioxidants, anticancer and antimicrobial agents [15]. In Europe and Caucasia, many cultures and civilizations have traditionally used wild edible macro-fungi for centuries in cooking, traditional medicine and other anthropogenic applications, a tendency which is increasingly more accepted [16]. Some macro-fungi belonging to the class of Basidiomycetes, such as the *Agaricus* species, which are hypoglycemic, antihyperlipidemic, antimicrobial, antioxidant, anticlastogenic, antitumor, antiangiogenic and wound healing; the *Agrocybe* species, which are anticarcinogenic, anti-inflammatory, antioxidant, antibacterial, antifungal and mitogenic and have antiproliferative properties; and the Ascomycetes, such as the edible basidiomycete macro-fungi, which have protein/enzyme, antimicrobial, antioxidant and nephroprotective activities [17,18]. 

Macro-fungi species exhibit great differences in morphological traits and biochemical content (i.e., antioxidant capacity and total phenolic contents), and the genera *Morchella* and *Agaricus* were found to have the highest value in terms of biochemical content. Thus, it is worth working with naturally grown macro-fungi species in terms of their biochemical content in different parts of the world to reach consensus on the biochemical content of the different genera. In a previous study, high contents of phenolic compounds could account for the good antioxidant properties in different macro-fungi species, and among them, *Leucopaxillus giganteus* had the highest content of total phenols [11]. In another study, the total phenolic and antioxidant properties of the *Pleurotus ostreatus* and *P. citrotinusus* macro-fungi produced in various wood dusts were determined, and significant differences were observed [2]. Several studies showed that the total antioxidant capacities and total phenolic contents of diverse macro-fungi species are quite variable, and strong relationships have been reported between total phenolic content and antioxidant capacity. These studies also indicated that macro-fungi have antimicrobial characteristics [19,20,21,22,23]. Therefore, macro-fungi could be used both as a functional food or ingredient in functional products.

In fact, horticultural plants including macro-fungi present diverse morphological traits and biochemical contents [24,25,26,27,28,29]. In addition, along with compositional differences among macro-fungi species, different plant parts of the same macro-fungi may exhibit compositional differences. Therefore, it is of great importance to identify not only the species level but also each edible part of the same mushroom species. 

The Van Lake region located in eastern Turkey is one of the most fascinating areas in terms of flora diversity and has a high wild edible macro-fungi diversity. Despite of the great popularity of the wild grown macro-fungi available in the region, data regarding their morphological traits and biochemical composition as well as their nutritional value are very scarce.

Therefore, in this study, we aimed to determine some biochemical (soluble solid contents, pH, total antioxidant capacity and total phenol) and morphological traits (stem length, stem width, cap length, cap width and color values) of different parts of some macro-fungi species naturally (wildly) grown at Van Lake region. In terms of the development of novel products and innovative value chains, particularly in the context of their healthy components, determination of the biochemical content of wild edible macro-fungi is a promising strategy for their further valorization. It is thought that the present study will contribute to future studies because the morphological traits and biochemical contents of the macro-fungi are scarce in the literature, and some species used in the present study have not been studied yet.

## 2. Materials and Methods

**Figure 1:**
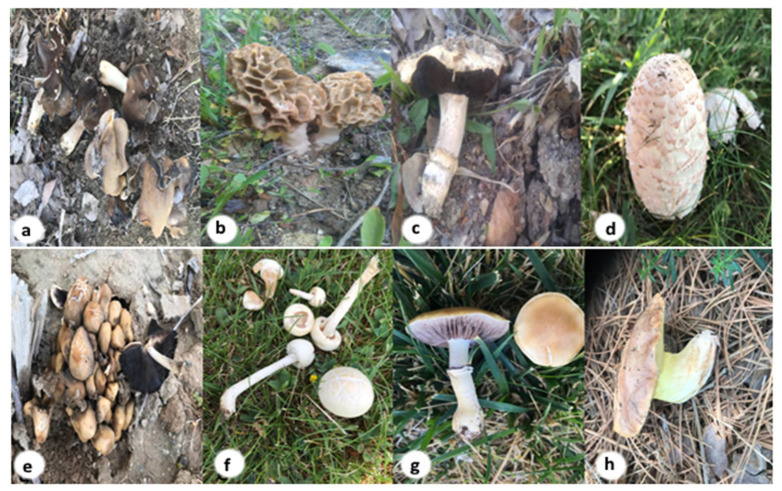
General view of all mushrooms collected: (**a**) *Helvella leucopus*, (**b**) *Morchella esculenta*, (**c**) *Agaricus bitorquis*, (**d**) *Coprinus comatus*, (**e**) *Coprinellus micaceus*, (**f**) *Agrocybe dura*, (**g**) *Psilocybe coronilla*, (**h**) *Suillus collinitus*.

The study materials were collected from the campus area of Van Yuzuncu Yil University (YYU) in April–May 2018. Macro-fungi samples were photographed in their habitats (Figure 1), and essential ecological and morphological properties were noted. The samples brought to the laboratory were kept fresh at −80 °C in the freezer (Esco Micro Pte Ltd. Model 363L, Singapore). Microscopic and macroscopic measurements and observations of macro-fungi were made by using the relevant literature in the laboratory [30,31,32,33]. Color indices, pH, soluble solid contents (SSC), total antioxidant capacity (TAC), and total phenolic contents (TPC) were examined in naturally grown eight macro-fungi species. All environment parameters were indicated below (Table 1). References [34,35] were used to identify samples.

### 2.1. Determination of Morphological Properties

#### 2.1.1. Width and Length of Macro-Fungi

Morphological measurements of macro-fungi were completed with the help of a digital compass and the parameters of stem length, stem width, cap length and cap width were noted in cm (±0.5).

#### 2.1.2. Color Indices (L*, a*, b*, C°, and h°)

Color indices were measured with the aid of a Minolta Color Meter (Model CR-400; Osaka, Japan) on the cap surface, cap basement and stems of macro-fungi, separately.

### 2.2. Determination of Biochemical Contents

#### 2.2.1. Total Phenolic Content and Total Antioxidant Capacity

Five grams of samples were taken separately from the stems and caps of mushrooms with different moisture contents; 25 mL of methanol was added and homogenized for 2 min with a Ultra Turrax model T25 basic homogenizer (IKA Works, Willmington, NC, USA) at medium speed and then exposed to dark conditions at room temperature for 30 min. The samples were filtered on Whatman no 1. filter paper and put into the Eppendorf tubes and stored at −80 °C until analysis. The total phenolic content was determined by spectrophotometer (Thermo Scientific Genesys 10S Model UV-VIS spectrophotometer Waltham, MA, USA) using the Folin–Ciocaltaeu colorimetric method [36]. The absorbance values of the solutions were read spectrophotometrically at 725 nm wavelength and the total phenolic content expressed as mg gallic acid equivalent (GAE) kg^−1^ fresh weight (FW). Ferric Reducing Antioxidant Power (FRAP) (Iron (III) reduction antioxidant power) method was used to determine antioxidant capacity [37]. The readings were taken at 593 nm in the absorbance spectrophotometer, and the antioxidant activity values were given as Trolox equivalent (TE) mg^−1^.

#### 2.2.2. pH

The pH values were determined through inserting the probe of a pH meter (Metler Toledo; Seven Compact pH/Ion S220 Colombus, OH, USA) into the juice of mushroom samples, and the homogenates were centrifuged at 4000 rpm for 40 min at 30 °C [38,39].

#### 2.2.3. Soluble Solids Contents (SSC)

The soluble solids content was measured with a digital refractometer (Atago, Tokyo, Japan) in mushroom juice; the homogenates were centrifuged at 4000 rpm for 40 min at 30 °C, and the results were expressed in ° Brix [38,39].

### 2.3. Statistical Analysis

One Way ANOVA was used to determine the difference between species. Duncan multiple comparison was used to determine the significant levels in species. Moreover, independent samples t test was used to determine the variation of different parts of the same species separately, and the statistical significance level was taken as 5% in the calculations. SAS 9.4 statistical program was used for all the required statistical analyzes.

## 3. Results and Discussion

Macro-fungi which were identified systematically were classified, and their biochemical contents were analyzed. The dimensional properties of the 8 macro-fungi were given below (Table 2), and the results of biochemical contents and color values were obtained from the macro-fungi of these sizes.

### 3.1. Determination of Morphological Properties

The length and width values of the eight different macro-fungi species collected in different periods were determined separately on cap and stem and given in Table 2. The stem length ranged from 3.00 cm to 16.00 cm. While the highest stem length (16.00 cm) was that of *C. comatus* followed by that of *A*. *bitorquis* species with 7.85 cm, the shortest length (3.00 cm) was found in *P. coronilla*. The stem width values varied from 0.35 cm to 4.75 cm, and the highest value (4.75 cm) was found in *C. comatus* followed by *A. bitorquis* species with 2.45 cm. These high values for stem width were determined to be the same as the top ranking stem lengths, and the lowest value (0.35 cm) was found in *C. micaceus*. While cap width values ranged from 1.90 cm to 9.25, cap length values ranged from 1.40 cm to 9.25 cm. The highest length value was found in *C. comatus* species with 9.25 cm, similar to the highest values of stem length and width. However, it was followed by *H. leucopus* and *M. esculenta* species with 5.05 cm and 4.05 cm, respectively, and the lowest cap length value was found in *P. coronilla* species with 1.40 cm. 

The highest width value was found in A. *bitorquis* species with 9.25 cm, and this value was followed by those of *C. comatus* and *S. collinitus* species with 9.00 cm and 7.05 cm, respectively, and the lowest cap width was found in *A. dura* species with 1.90 cm.

### 3.2. Determination of Color Indices

The color values (L*, a*, b*, C ˚and h˚) of the macro-fungi species were evaluated separately in cap surface, cap basement and stem and given in Table 3. Among these values, the L color values ranged from 21.28 to 78.09, and the highest L value was found in the cap surface part of *A. dura* with 78.09, and this value was followed by values found in the stem part of *C. comatus* and *P. coronilla* species as 74.98 and 70.04, respectively. The lowest L values were found on the cap basement parts of *A. bitorquis* and *C. micaceus* species with 21.28 and 28.47, respectively, and these values were followed by 30.46 measured on the cap surface of the *H. leucopus* species. It was concluded from the determined values that the lowest L values were found on cap basements. Therefore, when low values are evaluated for consumption in the basement of the macro-fungi, this does not pose a problem in terms of brightness, and this situation originates from the species-specific color. In addition, L values was found to be close to the optimum value ranges in all macro-fungi species. These macro-fungi also have a high source of phenolic contents. For this reason, they may serve as nutritious food in the human diet and it may help decrease the oxidative damage. It has been determined that dark colored mushrooms have higher phenolic content. Therefore, it can be concluded that *H. leucopus* and *M. esculenta* species, which are among the mushrooms grown in the region that have a darker color than the others, can be recommended to the consumers. Value a varied from −0.07 to 10.51, and the highest a value (10.51) was found on the cap surface of the *C. micaceus* species. The lowest a value was found on the stem part of *C. comatus* species with −0.07, and thus, the highest value for the blue color was found in this species. In terms of a color value, cap surface parts were higher than cap basement parts in all macro-fungi types outside of *A. dura* specie. Value b ranged from 2.07 to 43.58, and the highest b value (43.58) was found on the cap basement of *S. collinitus* species. The lowest b color value was measured as 2.07 at the cap basement part of *A. bitorquis* species, and this value was followed by 6.09 measured at the cap basement part of *C. micaceus* species. C value varied from 2.41 to 41.30, and the highest C value (41.30) was found on the cap basement part of the *S. collinitus* species. This value was followed by a value of 38.83 measured on the cap surface of the *P. coronilla* species. The lowest C color value was measured at 2.41 at the cap basement of the *A. bitorquis* species, and this value was followed by 7.16 measured at the cap basement of the *C. micaceus* species. In addition, the lowest values for b and C color parameters were found at the cap basements of the *A. bitorquis* and *C. micaceus* species. Value h ranged from 54.25 to 91.16, and the highest a value (91.16) was found on the stem of *C. comatus* species; this value was followed by values found on the stem of the A. dura species and the cap basement of *C. comatus* species at 86.60 and 85.11, respectively. The lowest h value was found on the cap basement of the *P. coronilla* species with 54.25, and this value was followed by the 55.00 and 55.54 values found on the cap basement of the *C. micaceus* species and on the cap surface of the *H. leucopus* species, respectively. There was a statistical difference between the different parts of the same species and between the same parts of different species (*p* < 0.01). There are limited detailed studies in literature on color traits in naturally grown mushrooms. Reference [40] studied the effect of cytokinin on the storage of white button mushrooms (*Agaricus bisporus*) and reported L, C, h color values at different day intervals, as L value 86.635, C value 16.665 and h value 83.725 at zero days.

### 3.3. Determination of Biochemical Contents

The biochemical parameters of the eight different macro-fungi species collected in different periods were measured separately in cap and stem and given in Table 4. It was found that all macro-fungi species were close to neutral (7.00) in pH value. In addition, these values varied from 6.62 to 8.75, and the highest pH value was found in the stem of *C. micaceus* with 8.75, and it was determined that this value was followed by the cap of same species with 8.52. It was found that the pH value of the cap of *C. micaceus* species was more acidic than the stem. However, this value was followed by 8.43 and 8.39, and these values were found in the cap and stem of the *C. comatus* species, respectively. It was also found that part of the stem was more acidic than part of the cap in contrast to the *C. micaceus* species. The lowest pH value was found in the stem of the S. *collinitus* species and was measured at 6.62, and it was also found that as with the *C. comatus* species, the stem was more acidic than the cap. In addition, in terms of pH value, the stems of *C*. *comatus*, *S*. *collinitus* and *P. coronilla* species were more acidic than the cap; however, the caps of the remaining five macro-fungi species had lower pH than the stems, and therefore, the caps were more acidic. While there was no statistical difference between the different parts of the same species, there was a difference between the same parts of different species (*p* < 0.01). In a study of thermally buffered corrugated packing pH of all fungi samples measured as 6.17 in 0 days and after 5 days of storage samples, the pH values were measured as 6.75, and this represented a significant change [23]. In a study conducted on mycelium cultures, researchers applied different temperature and pH experiments, and they suggested that the best pH = 6.5 and that at 25 °C mushrooms grow well [22].

The soluble solid content (SSC) values ranged from 2.25° brix to 5.80° brix, and the highest SSC value was found in part of the cap of *H. leucopus* species with 5.80° brix. It was determined that this value was followed by that of the stem of the *C. comatus* species with 5.05° brix. The lowest SSC value was found in the stem of *C. micaceus* species with 2.25° brix, and this value was followed by the 3.70° brix value measured in the stem part of *P. coronilla* species. In addition, the stem parts of the *C. comatus*, *A. dura* and S. *collinitus* species were higher than the cap parts, while the cap parts of the remaining macro-fungi species had a higher rate than the stem parts in terms of soluble solid content. It was also found that *H. leucopus*, *S. collinitus*, *C. comatus*, *A. bitorquis* and *M. esculenta* species have a higher percentage than other mushroom species from the point of stems and caps. Therefore, there was a statistical difference in the different parts of the same species in different ratios. The stem parts of all species showed statistically significant differences between each other (*p* < 0.01) for SSC. The cap parts of all species have also exhibited statistically significant differences between each other (*p* < 0.05) for SSC.

Total antioxidant capacity (TAC) values ranged from 15.72 µmol TE g^−1^ and 57.67 µmol TE g^−1^, and the highest TAC value was found in the cap part of *M. esculenta* species with 57.67 µmol TE g^−1^, and this value was followed by the stem part of *A. bitorquis* species with 54.81 µmol TE g^−1^. These values were followed by 46.17 µmol TE g^−1^ and 45,300 µmol TE g^−1^ found in the stem and cap parts of S. *collinitus* species, respectively. The lowest value for TAC was found in the stem of *C. comatus* species at 15.72 µmol TE g^−1^, and also, the TAC value of the cap part of the same species was found to be about double, the value being 27.77 µmol TE g^−1^. Moreover, it was found that the stem parts of *C. micaceus*, *A. bitorquis*, *A. dura* and *S*. *collinitus* species had more total antioxidant capacity than parts of the cap; however, the cap part of the remaining macro-fungi species had higher amounts of total antioxidant capacity than the stem parts. In the present study, TAC content of the cap part of *A. bitorquis* species was found as µmol TE g^−1^, and also TAC value of the stem part of same species was found to be about double the amount, the value being 54.81 µmol TE g^−1^. While the TAC content (57.67 µmol TE g^−1^) found in the cap part of *M. esculenta* species was much higher than the TAC content (34.42 µmol TE g^−1^) found in the stem part of *M. esculenta* species. Therefore, there was a statistical difference between the different parts of same species in different ratios and between the stem parts of different species (*p* < 0.05). In a study, *A*. *bisporus* was found to be the species with the lowest antioxidant activity (10% of inhibition) [41]. According to [42], regarding the nutritional composition and antioxidant capacity of several edible mushrooms grown in Southern Vietnam, the total bound phenolic content in the extract of *Ganoderma lucidum* was found to have a high antioxidant capacity. In Reference [43], conducted for the determination of total antioxidant content in 49 edible macro-fungi species, very different results were obtained from macro-fungi, and these ranged from 4.718 mmol Trolox per g (in *Tremella aurantialba* Zang) to 43.178 mmol Trolox per g (in *Volvariella volvacea* Sing). Reference [19] studied total antioxidant capacity of 12 macro-fungi species collected from different regions and reported that their antioxidant capacity varied from 525.32 µmol TE 100 g^−1^ (in *Lactarius semisanguifluuss*) to 1693.85 µmol TE 100 g^−1^ (in *Hydnum repandum*).

Total phenolic content (TPC) values varied from 13.85 mg GAE kg^−1^ FW to 60.16 mg GAE kg^−1^ FW, and the highest TPC value was found in the stem part of *M. esculenta* species with 60.16 mg GAE kg^−1^ FW, and this value was followed by the cap part of *S. collinitus* (59.91 mg GAE kg^−1^ FW) and the cap part of *H. leucopus* (mg GAE kg^−1^ FW). The lowest value for TPC was found to be 13.85 mg GAE kg^−1^ FW and 17.91 mg GAE kg^−1^ FW in the stem parts of *C. comatus* and *P. coronilla* species, respectively. Moreover, it was found that the stem parts of *M. esculenta* and *A. bitorquis* species had more total phenolic content than parts of the cap; however, the caps of the remaining six macro-fungi species had higher amounts of total phenolic content than the stem parts. In addition, in terms of TAC and TPC content, it was found that *M. esculenta*, *S. collinitus*, *H. leucopus* and *A. bitorquis* species had higher values than the remaining four macro-fungi species for the stem and cap parts. In addition, there was statistical difference in the different parts of same species and between the stem and cap parts of different species (*p* < 0.01). 

Researchers studying different plant species reported that ripe fruit flesh contains 10 times more phenol than unripe fruit flesh [44,45]. In Reference [41], examining the total phenolic content of edible fungi, *Boletus edulis* (≈5.5) had the highest content in dried fungi samples, followed by *Agaricus bisporus* (≈3.5). According to [46], the total phenolic contents were found to be 6.60 mg of GAEs g^−1^ of dry mushroom for *Lentinula edodes* and 17.0 mg of GAE g^−1^ of dry mushroom for *Volvariella volvacea*. In a study conducted on macro-fungi, the total phenol contents varied from each other depending on extraction solvent and used material. Therefore, these contents were found to be 109.35–221.77 µg GAE mg^−1^ extract for *Roccella phycopsis* ethanol extract, 106.55–212.27 mg GAE mg^−1^ extract for *R. phycopsis* methanol extract, 62.44–119.85 µg for *Flavoparmelia caperata* ethanol extract and 63.5–170.14 µg GAE mg^−1^ for *F. caperata* methanol extract. In a study of five different *Agaricus* species, total phenol content varied from 2.72 to 8.95 mg g^−1^ [47]. In a study of three different mushroom species (*Leucopaxillus giganteus*, *Sarcodon imbricatus* and *Agaricus arvensis*), total phenol contents were found to be 6.29 mg g^−1^ 3.76 mg g^−1^ and 2.83 mg g^−1^, respectively [11]. In another study, total phenolic contents of 12 macro-fungi species collected from different regions were investigated and total phenolic contents varied from 575.10 mg DW 100 g^−1^ to 2156.40 mg DW 100 g^−1^ [19]. Previous studies conducted on different horticultural crops showed great differences among used samples for total phenolic content [48,49,50,51,52,53].

## 4. Conclusions

In the present study, macro-fungi collected from different locations and habitats of the Van lake area were identified. The cap parts of the mushrooms are usually consumed. Therefore, considering the cap parts of the mushrooms in light of the current data evaluated in terms of soluble solid content, *H. leucopus, M. esculenta, A. bitorquis* and *S. collinitus* came to the fore, respectively. Considering the total antioxidant capacity, *M. esculenta, P. coronilla and S. collinitus* have exhibited higher values than the others. In terms of total phenolic content, *S. collinitus*, *H. leucopus* and *M. esculenta* were found to be its richest source. When all mushrooms were evaluated together, the most remarkable results were obtained from the *M. esculenta*, because both the stem and cap parts are consumed together, and it has expressed the highest biochemical content. The characterization of these wild edible macro-fungi species from the Van lake region represent ground data for further studies related to the possibilities and sustainability of their use in developing new functional products and/or ingredients.

## Figures and Tables

**Table 1:** Macro-fungi used in the study.

Division	Species	Collected Area and Habitat	Moisture Content (%)	Coordinates	Altitude	Collected Date	Sample No
*Ascomycota*	*Helvella leucopus* Pers.	Campus area, entrance of Horticulture department experimental area, under *Populus* sp.	85.50	38°34′10″ K; 43°17′65″ D	1672 m	17 May 2018	ALP.7
	*Morchella esculenta* (L.) Pers	Campus area, near the Faculty of Veterinary Medicine, Transformer adjacent, on meadow	78.00	38°34′26″ K; 43°16′58″ D	1660 m	4 May 2018	ALP.5
*Basidiomycota*	*Agaricus bitorquis* (Quél.) Sacc.	Campus area over hospital road, meadow area	92.00	38°34′14″ K; 43°17′57″ D	1673 m	2 May 2018	ALP.8
	*Coprinus comatus* (O.F. Müll.) Pers	Campus area, garden of Agricultural Faculty, meadow area	90.50	38°34′02″ K; 43°16′36″ D	1660 m	24 May 2018	ALP.1
	*Psilocybe coronilla* (Bull.) Noordel.	Campus area mosque backyard, meadow area	80.00	38°34′78″ K; 43°16′05″ D	1666 m	19 May 2018	ALP.2
	*Coprinellus micaceus* (Bull.) Vilgalys, Hopple & Jacq. Johnson	Campus area Agricultural Faculty garden, *Salix* sp. adjacent	88.50	38°34′38″ K; 43°16′73″ D	1661 m	15 May 2018	ALP.3
	*Agrocybe dura* (Bolton) Singer	Campus area mosque backyard, meadow area	86.00	38°34′44″ D; 43°16′21″ D	1667 m	15 May 2018	ALP.4
	*Suillus collinitus* (Fr.) Kuntze	Campus area near the mosque, under *Pinus* sp.	95.30	38°34′15″ K; 43°17′14″ D	1672 m	17 May 2018	ALP.6

**Table 2:** Determination of length and width sizes in some naturally grown mushroom species.

Species	Stem Length (cm)	Cap Length (cm)	Stem Width (cm)	Cap Width (cm)
*Helvellaa leucopus*	4.50	5.05	1.55	5.15
*Morchella esculenta*	4.90	4.05	1.60	5.10
*Agaricus bitorquis*	7.85	3.10	2.45	9.25
*Coprinus comatus*	16.00	9.25	4.75	9.00
*Coprinellus micaceus*	5.80	2.20	0.35	2.85
*Agrocybe dura*	5.20	1.50	0.45	1.90
*Psilocybe coronilla*	3.00	1.40	1.00	5.60
*Suillus collinitus*	5.85	3.05	2.10	7.05

**Table 3:** Determination of color values (L, a, b, C, h) in naturally grown some macro-fungi species.

Species	Investigated Parts	L *	a *	b *	C°	h°
*Helvella leucopus*	Cap surface	30.46 C ** (d **)	6.15 A ** (ab)	9.42 B ** (e **)	11.30 B * (e **)	55.54 B ** (d **)
	Cap basement	52.98 B ** (bc **)	1.26 C ** (c **)	10.11 B ** (cd **)	10.19 B * (bcd **)	82.95 A ** (a **)
	Stem	64.29 A ** (ab **)	3.11 B ** (cd **)	23.41 A ** (bc **)	23.64 A * (c **)	82.52 A ** (bc **)
*Morchella esculenta*	Cap surface	37.42 B * (d **)	6.43 A * (ab)	19.22 A (d **)	20.28 A (d **)	71.58 A (bc **)
	Cap basement	51.31 A * (c **)	4.34 B * (b **)	17.86 A (bc **)	18.39 A (bc **)	76.28 A (a **)
	Stem	57.68 A * (bc **)	4.20 B * (bc **)	19.25 A (cd **)	19.72 A (cd **)	74.73 A (d **)
*Agaricus bitorquis*	Cap surface	56.17 A ** (c **)	8.69 A ** (a)	27.21 A ** (bc **)	28.57 A ** (bc **)	72.25 A ** (bc **)
	Cap basement	21.28 B ** (e **)	1.23 B ** (c **)	2.07 B ** (d **)	2.41 B ** (d **)	58.45 B ** (b **)
	Stem	44.69 A ** (d **)	7.84 A ** (a **)	20.45 A ** (bc **)	22.00 A ** (c **)	68.66 A ** (d **)
*Coprinus comatus*	Cap surface	58.18 A (c **)	6.05 A ** (ab)	19.67 A * (d **)	20.59 A * (d **)	72.96 B * (abc **)
	Cap basement	69.30 A (a **)	0.73 B ** (c **)	9.49 B * (cd **)	9.54 B * (bcd **)	85.11 A * (a **)
	Stem	74.98 A (a **)	−0.07 B ** (e **)	11.30 B * (de **)	11.32 B * (e **)	91.16 A * (a **)
*Coprinellus micaceus*	Cap surface	53.20 A ** (c **)	10.51 A ** (a)	29.89 A * (b **)	31.70 A ** (b **)	70.61 AB (bc **)
	Cap basement	28.47 B ** (de **)	3.67 B ** (b **)	6.09 B * (cd **)	7.16 C ** (cd **)	55.00 B (b **)
	Stem	48.31 A ** (cd **)	4.32 B ** (bc **)	7.35 B * (e **)	15.96 B ** (d **)	74.24 A (d **)
*Agrocybe dura*	Cap surface	78.09 A * (a **)	2.60 A (b)	23.40 A (cd **)	23.60 A (cd **)	84.21 A (a **)
	Cap basement	63.19 B * (ab **)	4.10 A (b **)	24.21 A (b **)	24.57 A (b **)	80.27 A (a **)
	Stem	65.38 B * (ab **)	0.97 A (de **)	16.16 A (cd **)	16.19 A (d **)	86.60 A (ab **)
*Psilocybe coronilla*	Cap surface	68.03 A * (b **)	6.00 A (ab)	39.81 A ** (a **)	38.83 A ** (a **)	81.39 A * (ab **)
	Cap basement	36.24 B * (d **)	5.41 A (a **)	8.31 C ** (cd **)	10.10 B ** (bcd **)	54.25 B * (b **)
	Stem	70.04 A* (a **)	2.95 A (cd **)	33.94 B ** (a **)	34.15 A ** (a **)	85.04 A * (ab **)
*Suillus collinitus*	Cap surface	39.60 A (d **)	8.87 A (a)	19.17 B (d **)	21.32 A (d **)	65.80 A (cd **)
	Cap basement	51.08 A (c **)	6.01 A (a **)	43.58 A (a **)	41.30 A (a **)	83.69 A (a **)
	Stem	55.17 A (bcd **)	6.62 A (ab **)	28.38 AB (ab **)	29.38 A (b **)	75.96 A (cd **)

* *p* < 0.05, ** *p* < 0.01. The lowercase written in parentheses shows statistical differences between cap surface, cap basement and stem part of different species. The uppercase written in the table shows statistically differences between same species of different plant parts.

**Table 4:** Determination of pH, soluble solid contents (SSC °Brix), total antioxidant capacity (TAC µmol TE g^−1^ FW) and total phenolic contents (TPC mg GAE kg^−1^ FW) in some naturally grown mushroom species. (FW = Fresh Weight, GAE = Gallic Acid Equivalent, TE = Trolox Equivalent).

Species	Investigated Parts	pH	SSC	TAC	TPC
*Helvella leucopus*	Stem	8.06 A (c **)	4.55 B * (ab **)	27.42 A (bc *)	47.72 A (b **)
	Cap	7.97 A (bc **)	5.80 A * (a *)	34.17 A (ab)	57.47 A (ab **)
*Morchella esculenta*	Stem	7.65 A (d **)	4.35 A (ab **)	34.42 B ** (abc *)	60.16 A (a **)
	Cap	7.60 A (d **)	4.95 A (ab *)	57.67 A ** (a)	46.79 A (bc **)
*Agaricus bitorquis*	Stem	7.82 A (d **)	4.40 A (ab **)	54.81 A * (a *)	19.22 B ** (c **)
	Cap	7.80 A (cd **)	4.95 A (ab *)	22.92 B * (b)	33.29 A ** (de **)
*Coprinus comatus*	Stem	8.39 A (b **)	5.05 A (a **)	15.72 B * (c *)	13.85 B ** (c **)
	Cap	8.43 A (a **)	4.35 A (b *)	27.77 A * (b)	44.16 A ** (cd **)
*Coprinellus micaceus*	Stem	8.75 A (a **)	2.25 B ** (c **)	20.39 A (c *)	22.10 A (c **)
	Cap	8.52 A (a **)	3.95 A ** (b *)	19.35 A (b)	26.22 A (ef **)
*Agrocybe dura*	Stem	8.15 A (c **)	4.50 A (ab **)	25.35 A (bc *)	21.66 A (c **)
	Cap	8.08 A (b **)	3.95 A (b *)	21.06 A (b)	21.54 A (ef **)
*Psilocybe coronilla*	Stem	7.58 A (d **)	3.70 A (b **)	27.06 B ** (bc *)	17.91 A (c **)
	Cap	7.69 A (d **)	3.90 A (b *)	35.02 A ** (ab)	18.60 A (f **)
*Suillus collinitus*	Stem	6.62 A (e **)	4.90 A (ab **)	46.17 A (ab *)	51.35 A (ab **)
	Cap	6.84 A (e **)	4.65 A (ab *)	45.30 A (ab)	59.91 A (a **)

* *p* < 0.05, ** *p* < 0.01. The uppercase written in the table shows statistical differences within different parts of the same species. The lowercase written in the table shows statistically differences between the same parts of different species.

## Data Availability

All new research data were presented in this contribution.
